# The cost-effectiveness of a school-based overweight program

**DOI:** 10.1186/1479-5868-4-47

**Published:** 2007-10-01

**Authors:** Henry Shelton Brown, Adriana Pérez, Yen-Peng Li, Deanna M Hoelscher, Steven H Kelder, Roberto Rivera

**Affiliations:** 1 Division of Management, Policy and Community Health, University of Texas School of Public Health, Austin, TX 78701, USA; 2 Department of Bioinformatics and Biostatistics School of Public Health and Information Sciences University of Louisville 555 S. Floyd Street, Suite 4026 Louisville, KY 40292, USA; 3 Division of Biostatistics, University of Texas School of Public Health, Houston, TX 77225, USA; 4 Division of Behavioral Science, University of Texas School of Public Health, Austin, TX 78701, USA; 5 Division of Epidemiology, University of Texas School of Public Health, Austin, TX 78701, USA; 6 Valley Baptist Hospital, Harlingen, TX 78520, USA; 7 Michael & Susan Dell Center for Advancement of Healthy Living, University of Texas School of Public Health, Austin, TX 78701, USA

## Abstract

**Background:**

This study assesses the net benefit and the cost-effectiveness of the Coordinated Approach to Child Health (CATCH) intervention program, using parameter estimates from the El Paso trial. There were two standard economic measures used. First, from a societal perspective on costs, cost-effectiveness ratios (*CER*) were estimated, revealing the intervention costs per quality-adjusted life years (*QALY*s) saved. *QALY *weights were estimated using National Health Interview Survey (NHIS) data. Second, the net benefit (*NB*) of CATCH was estimated, which compared the present value of averted future costs with the cost of the CATCH intervention. Using National Health and Nutrition Examination Survey I (NHANES) and NHANES follow-up data, we predicted the number of adult obesity cases avoided for ages 40–64 with a lifetime obesity progression model.

**Results:**

The results show that CATCH is cost-effective and net beneficial. The *CER *was US$900 (US$903 using Hispanic parameters) and the *NB *was US$68,125 (US$43,239 using Hispanic parameters), all in 2004 dollars. This is much lower than the benchmark for *CER *of US$30,000 and higher than the *NB *of US$0. Both were robust to sensitivity analyses.

**Conclusion:**

Childhood school-based programs such as CATCH are beneficial investments. Both *NB *and *CER *declined when Hispanic parameters were included, primarily due to the lower wages earned by Hispanics. However, both *NB *and *CER *for Hispanics were well within standard cost-effectiveness and net benefit thresholds.

## Background

Childhood overweight is a major threat to child health in the US [1]. Unfortunately, overweight children are not likely to return to normal weight later in life [2-4]. Aside from the correlation of lifetime behaviors [5], treatment strategies for obese adults remain largely ineffective [6-11]. Obesity in adulthood is closely associated with chronic diseases including cardiovascular disease (CVD), type 2 diabetes, high blood pressure, stroke, high blood cholesterol levels, joint problems, some cancers, and gall bladder disease [12-15]. The prevalence of overweight [1] among children has doubled in the last twenty years [16], disproportionately affecting minorities [17-20].

Because no other institution has as much continuous and intensive contact with children, schools can provide a pivotal role in physical activity and nutrition interventions. Further, school programs can be delivered at low cost to families, reaching all socioeconomic levels. A number of school-based interventions aimed at promoting healthy behaviors have been evaluated for effectiveness in terms of outcomes in the last 15 years [21-30]. Of all these programs, two stand out among the rest because of their sophisticated study design (Coordinated Approach to Child Health (CATCH)) and program impact on childhood overweight (Planet Health). Given that there are relatively few dollars for overweight prevention, comparisons between alternative prevention programs are warranted [31].

If childhood overweight prevalence is reduced and this in turn reduces adulthood obesity, there will be large economic benefits [32,33]. For instance, one study estimates that obesity costs were US$99.2 billion in 1995 [34]. Indirect costs include labor productivity due to obesity [31,35] and co-morbidities such as diabetes, which in themselves is negatively related to working propensity [36-38]. Second, direct, or medical, costs are higher [39].

In this economic evaluation of CATCH, we focused on adulthood obesity which results from child overweight, the period of life where costs of obesity are higher. There were two economic measures. First, from a societal perspective of costs, cost-effectiveness ratios (*CER*) were estimated. *CER *provided the cost per quality-adjusted life years (*QALY*s) saved. Second, the net benefit (*NB*) of CATCH was estimated. *NB *compared averted medical and labor productivity costs to the cost of the CATCH intervention.

### The CATCH program and the El Paso trial

During the years 2000–2002, there was a controlled trial of CATCH in El Paso, Texas [40-42]. The CATCH program trial followed a cohort of children across grades three, four, and five. In the U.S., most children start the third grade at age 8 and finish the fifth grade at age 11. The CATCH intervention program in the trial was identical to the national program [40-42]. The program components included a classroom curriculum at each grade level, a physical education program, modifications to the school food service, and family- and home-based programs. CATCH field staff conducted one day training for each of the intervention schools, with periodic on-site follow-up and mentoring over the three year period.

Four intervention schools and four matched control schools were randomly selected out of the two largest school districts in El Paso [40]. The control schools had 473 participants, composed of 224 girls and 249 boys. The intervention schools had 423 participants, composed of 199 girls and 224 boys. Over the three years, overweight and at-risk of overweight prevalence (at or above the 85^*th *^percentile of body mass index (BMI (weight in kilograms divided by height in meters squared *kg*/*m*^2^)) for sex and age) increased by 1% for boys and 2% for girls in the CATCH intervention schools, but increased by 9% for boys and 13% for girls in the control schools. Height and weight measures, used to calculate BMI were recorded in each of the three years during November, December, January, or February [40]. Quality of the anthropometry measures was maintained by comparing the average of each research assistant's measurements of height, weight, triceps skinfold and waist and hip circumference for research assistant with the trainer's measurement. For each random sample of participants used in the quality checks, three sets of measurements were made by each research assistant and compared to the trainer's measurements. Research assistants whose measures differed significantly were not allowed to continue.

Among the participating schools, 93% of the students were Hispanic [40]. As is the case for most border communities, English proficiency was not universal, ranging from 33% proficiency to 72% among the eight participating schools (intervention and control) [40]. Therefore, this study allows us to examine an overweight intervention in a culturally Hispanic, Mexican-American setting.

## Methods

**Figure 1:**
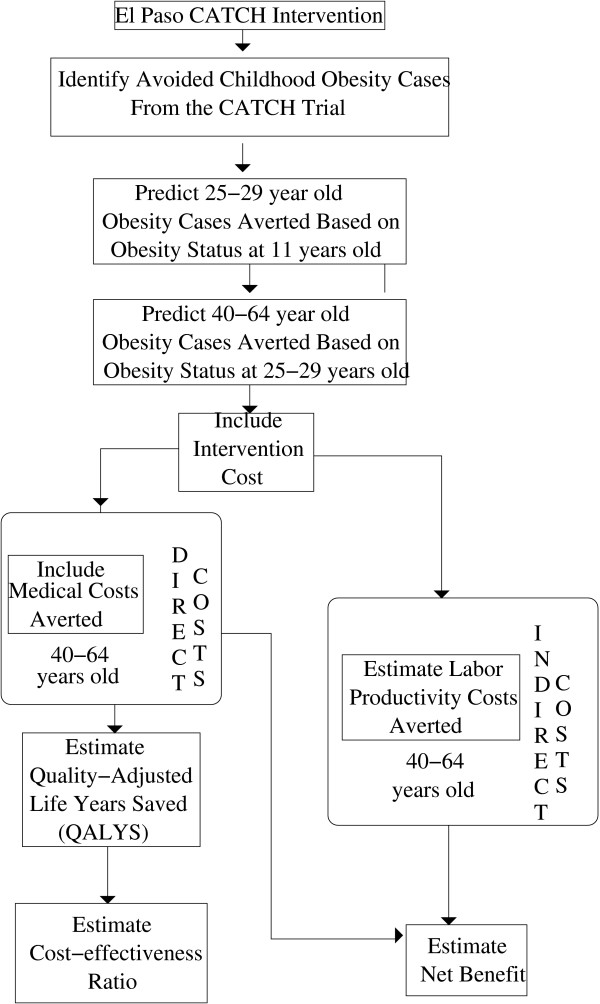
Flow Chart.

Our methods were similar to Wang *et al*. [35] A societal approach to costs was used as was a three percent annual discount rate. The flow chart in Figure 1 outlines the approach. First, we predicted the number of obese adult cases averted, as described in more detail below. Then we estimated costs associated with obesity and quality adjusted life-years beyond the age of 40. Note that labor productivity costs, medical costs and *QALY*s were relevant for cost-effectiveness ratios (*CER*); labor productivity costs and medical costs were relevant for net benefits (*NB*).

Let us first examine *CER*. The numerator of the *CER *is the cost of the intervention less the total medical costs due to obesity (which are averted due to the intervention). The medical costs are known as direct costs, and they would have been expected to have been incurred by society had the obese cases not been averted. In the denominator are total *QALY*s gained.

The *CER *formula is

CER=(C−∑iNi×Ai)/∑iNi×Qi,     (1)
 MathType@MTEF@5@5@+=feaafiart1ev1aaatCvAUfKttLearuWrP9MDH5MBPbIqV92AaeXatLxBI9gBaebbnrfifHhDYfgasaacH8akY=wiFfYdH8Gipec8Eeeu0xXdbba9frFj0=OqFfea0dXdd9vqai=hGuQ8kuc9pgc9s8qqaq=dirpe0xb9q8qiLsFr0=vr0=vr0dc8meaabaqaciaacaGaaeqabaqabeGadaaakeaacqWGdbWqcqWGfbqrcqWGsbGucqGH9aqpcqGGOaakcqWGdbWqcqGHsisldaaeqbqaaiabd6eaonaaBaaaleaacqWGPbqAaeqaaaqaaiabdMgaPbqab0GaeyyeIuoakiabgEna0kabdgeabnaaBaaaleaacqWGPbqAaeqaaOGaeiykaKIaei4la8YaaabuaeaacqWGobGtdaWgaaWcbaGaemyAaKgabeaakiabgEna0kabdgfarnaaBaaaleaacqWGPbqAaeqaaaqaaiabdMgaPbqab0GaeyyeIuoakiabcYcaSaaa@4C4D@

where subscript *i *= *m*, *f *indicates male and female, respectively. *C *represents the costs of the CATCH intervention in 2004 dollars, *N*_*i *_represents the number of adult obese cases averted due to CATCH, *A*_*i *_represents the averted medical costs when obese adults aged 40–64, inclusive, are instead non-obese adults; *Q*_*i *_represents the additional *QALY*s gained when obese adults are instead non-obese. The denominator is the additional *QALY*s accruing to averted obese adults due to the CATCH intervention. If the *CER *is less than approximately US$30,000, then we can consider the intervention cost-effective [43-45]. This is based on valuing a year of full human life at US$30,000. Other valuations of life-years are 10-fold this amount [46].

Now let us define net benefits (*NB*). We subtracted the intervention costs from the total averted medical costs and productivity costs between age 40 and 64, inclusive, for an average obese adult in comparison to an average non-obese adult. The *NB *formula is

NB=∑iNi×Ai+∑iNi×Bi−C,     (2)
 MathType@MTEF@5@5@+=feaafiart1ev1aaatCvAUfKttLearuWrP9MDH5MBPbIqV92AaeXatLxBI9gBaebbnrfifHhDYfgasaacH8akY=wiFfYdH8Gipec8Eeeu0xXdbba9frFj0=OqFfea0dXdd9vqai=hGuQ8kuc9pgc9s8qqaq=dirpe0xb9q8qiLsFr0=vr0=vr0dc8meaabaqaciaacaGaaeqabaqabeGadaaakeaacqWGobGtcqWGcbGqcqGH9aqpdaaeqbqaaiabd6eaonaaBaaaleaacqWGPbqAaeqaaOGaey41aqRaemyqae0aaSbaaSqaaiabdMgaPbqabaaabaGaemyAaKgabeqdcqGHris5aOGaey4kaSYaaabuaeaacqWGobGtdaWgaaWcbaGaemyAaKgabeaakiabgEna0kabdkeacnaaBaaaleaacqWGPbqAaeqaaOGaeyOeI0Iaem4qamealeaacqWGPbqAaeqaniabggHiLdGccqGGSaalaaa@4971@

where subscript *i *= *m*, *f *indicates male and female, respectively. *B*_*i *_represents the value of labor productivity gains for adults who have averted obesity.

In equations (1) and (2), *N*_*i *_is predicted from data from the obesity progression model, as described below [40].

### The intervention costs of CATCH

Intervention costs are given in Table 1. As is standard in economics, the value of the training time is the hourly wage. Wage and salary information for CATCH staff was suppressed for confidentiality. All wages are in 2004 US$.

**Table 1:** Intervention Costs, 2004 US$

CATCH Trainers			
Trainer	Notes	Hours	Cost

PE Specialist	Simultaneous training with PE Teachers (see below)	192	*
Classroom Specialist	Simultaneous training with Teachers (see below)	64	*
Eat Smart Nutrition Specialist	Simultaneous training with Food Specialist (see below)	192	*
Subtotal			US$7,815

Teacher Training Costs			

Trainee		Hours	Cost

Subject teachers†	2 teachers for 3 grades at 4 schools receive 8 hours summer training	192	US$3,615
PE teachers	1 PE teacher at 4 schools receives 8 hours summer training	192	US$3,615
Parent/nurse/counselor	1 parent or counselor at 4 schools receives 8 hours summer training	96	US$1,808
School Food	1 food specialist for 3 grades at 4 8 hours summer training	192	US$3,615
			US$12,654
Promotional Cost	4 schools		US$14,000
Total Cost			US$44,038

*Suppressed for confidentiality

†Each grade in all 4 schools must have a minimum of 2 teachers trained

Note that as in Wang *et al*., we excluded classroom time from the intervention cost [35]. CATCH increases the effectiveness of PE and classroom time without taking additional time away from other activities.

### Predicting adulthood obesity based on child overweight

We used the Centers for Disease Control and Prevention (CDC) definitions of child at-risk of overweight (85^*th *^percentile ≤ BMI ≤ 95^*th *^percentile for sex and age ) and child overweight (BMI > 95^*th *^percentile for sex and age). Henceforth, at-risk of overweight will be referred to as at-risk.

**Figure 2:**
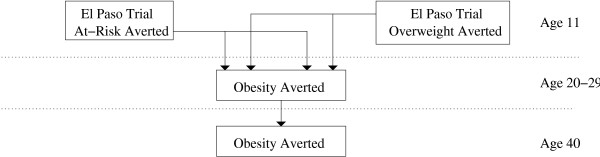
Projecting Adulthood Obesity.

The number of *adult *obese cases, defined as having a *BMI *> 30*kg*/*m*^2^, averted cannot be observed from the trial because it ends in the fifth grade. We used a lifetime obesity progression model to estimate averted adulthood obesity. The process is outlined in Figure 2.

Our lifetime obesity progression model is

Ni=Hi×(P5i−P6i)×∑j((P2ij5−P2ij3)−(P1ij5−P1ij3))×(P3ij−P4ij),     (3)
 MathType@MTEF@5@5@+=feaafiart1ev1aaatCvAUfKttLearuWrP9MDH5MBPbIqV92AaeXatLxBI9gBaebbnrfifHhDYfgasaacH8akY=wiFfYdH8Gipec8Eeeu0xXdbba9frFj0=OqFfea0dXdd9vqai=hGuQ8kuc9pgc9s8qqaq=dirpe0xb9q8qiLsFr0=vr0=vr0dc8meaabaqaciaacaGaaeqabaqabeGadaaakeaafaqaceGabaaabaGaemOta40aaSbaaSqaaiabdMgaPbqabaGccqGH9aqpcqWGibasdaWgaaWcbaGaemyAaKgabeaakiabgEna0kabcIcaOiabdcfaqnaaBaaaleaacqaI1aqncqWGPbqAaeqaaOGaeyOeI0Iaemiuaa1aaSbaaSqaaiabiAda2iabdMgaPbqabaGccqGGPaqkcqGHxdaTaeaadaaeqbqaaiabcIcaOiabcIcaOiabdcfaqnaaBaaaleaacqaIYaGmcqWGPbqAcqWGQbGAcqaI1aqnaeqaaOGaeyOeI0Iaemiuaa1aaSbaaSqaaiabikdaYiabdMgaPjabdQgaQjabiodaZaqabaGccqGGPaqkaSqaaiabdQgaQbqab0GaeyyeIuoakiabgkHiTiabcIcaOiabdcfaqnaaBaaaleaacqaIXaqmcqWGPbqAcqWGQbGAcqaI1aqnaeqaaOGaeyOeI0Iaemiuaa1aaSbaaSqaaiabigdaXiabdMgaPjabdQgaQjabiodaZaqabaGccqGGPaqkcqGGPaqkcqGHxdaTcqGGOaakcqWGqbaudaWgaaWcbaGaeG4mamJaemyAaKMaemOAaOgabeaakiabgkHiTiabdcfaqnaaBaaaleaacqaI0aancqWGPbqAcqWGQbGAaeqaaOGaeiykaKIaeiilaWcaaaaa@7460@

where subscript *i *= *m*, *f *again indicates male and female, respectively, and *j *= *a*, *o *represent at-risk or overweight. *N*_*i *_was defined above and *H*_*i *_represents the number of children in the fifth grade trial schools in El Paso [40]. *P*_2*ij*3 _and *P*_2*ij*5 _are the proportions of at-risk and overweight children in grades three (the beginning of the trial) and five (the end of the trial) in the control schools; *P*_1*ij*3 _and P_1*ij*5 _are the proportions of at-risk and overweight children in grades three and five in the intervention schools. *P*_3*ij *_captures the probabilities of obesity at age 21 to 29 conditional on being at-risk and conditional on being obese at age 11; *P*_4*ij *_measures the probabilities of obesity at age 21 to 29 conditional on being not at-risk and conditional on being not obese at age 11. *P*_5*i *_is the probability of obesity at age 40 conditional on being obese at age 21 to 29; *P*_6*i *_is the probability of obesity at age 40 conditional on not being obese at age 21 to 29.

### Data

Table 2 lists the conditional probabilities needed in (3) in expanded form along with their sources.

**Table 2:** Conditional Probabilities Needed for Predicting Adulthood Obesity

Women		
Type	Proportion	Source

*P*_1*fa*5 _= P(at-risk–intervention)	0.17	[40]
*P*_1*fa*3 _= P(at-risk–intervention)	0.17	[40]
*P*_1*fo*5 _= P(overweight–intervention)	0.15	[40]
*P*_1*fo*3 _= P(overweight–intervention)	0.13	[40]
*P*_2*fa*5 _= P(at-risk–no intervention)	0.21	[40]
*P*_2*fa*3 _= P(at-risk–no intervention)	0.09	[40]
*P*_2*fo*5 _= P(overweight–no intervention)	0.18	[40]
*P*_2*fo*3 _= P(overweight–no intervention)	0.17	[40]
*P*_3*fa *_= P(obese at 21–29y/o – at-risk at 11)	0.69	[9]
*P*_3*fo *_= P(obese at 21–29y/o – overweight at 11)	0.83	[9]
*P*_4*fa *_= P(obese at 21–29y/o – not at-risk at 11)	0.13	[9]
*P*_4*fo *_= P(obese at 21–29y/o – not overweight at 11)	0.16	[9]
*P*_5*f *_= P(obese at 40 y/o –obese at 25–29 y/o)	0.85	*
*P*_6*f *_= P(obese at 40 y/o –not obese at 25–29 y/o)	0.12	*

Men		

*P*_1*ma*5 _= P(at-risk–intervention)	0.14	[40]
*P*_1*ma*3 _= P(at-risk–intervention)	0.18	[40]
*P*_1*mo*5 _= P(overweight–intervention)	0.27	[40]
*P*_1*mo*3 _= P(overweight–intervention)	0.22	[40]
*P*_2*ma*5 _= P(at-risk–no intervention)	0.18	[40]
*P*_2*ma*3 _= P(at-risk–no intervention)	0.18	[40]
*P*_2*mo*5 _= P(overweight–no intervention)	0.31	[40]
*P*_2*mo*3 _= P(overweight–no intervention)	0.23	[40]
*P*_3*ma *_= P(obese at 21–29y/o – at-risk at 11)	0.69	[9]
*P*_3*mo *_= P(obese at 21–29y/o – overweight at 11)	0.83	[9]
*P*_4*ma *_= P(obese at 21–29y/o – not at-risk at 11)	0.13	[9]
*P*_4*mo *_= P(obese at 21–29y/o – not overweight at 11)	0.16	[9]
*P*_5*m *_= P(obese at 40 y/o –obese at 25–29 y/o)	0.77	*
*P*_6*m *_= P(obese at 40 y/o –not obese at 25–29 y/o)	0.12	*

[40]Coleman KJ, Tiller CL, Sanchez J, Heath EM, Sy O, Milliken G, Dzewaltowski DA: **Prevention of the Epidemic Increase in Child Risk of Overweight in Low-Income Schools: The El Paso Coordinated Approach to Child Health (El Paso)**. *Archives of Pediatrics & Adolescent Medicine *2005, 159:217–224.

[9]Whitaker RC, Wright JA, Pep e MS, Seidel KD, Dietz WH: **Predicting obesity in young adulthood from childhood and parental obesity. ***The New England Journal of Medicine *1997, 337(13):869–873.

Estimated by the authors. [47]

*Calculated from NHANES I Epidemiologic Followup Study (NHEFS) with 1975 National Health and Nutrition Examination Survey (NHANES) I data. [47]

In order to estimate the probability of obesity at age 40 conditional on being obese during ages 21–29, we linked 1992, 1987, and 1982 NHANES I Epidemiologic Followup Study (NHEFS) data with the original 1975 National Health and Nutrition Examination Survey (NHANES) I data [47]. For the 1975 data, BMI is available by sex and age. We kept those aged 25–29 from the 1975 data. Whichever follow-up dataset placed the subject closest to 40 was used. Those aged 28 and 29 in 1975 were linked to 1987 data (they were 40 and 41 then); those aged 25–27 in 1975 were linked to 1992 data (they were aged 42–44 then). The 'svy' facility of STATA 7.0© was used to account for the complex sampling design of NHANES. Note that Wang *et al*. use the same technique, but for females only [35].

### Medical costs averted (direct costs)

As in Wang *et al*., we used medical costs parameters from the literature [35].

### Data

Wang *et al*. used medical cost data for obese women between 40–64 years of age, inclusive, from Gorsky [48]. However, unlike in the Planet Health trial Wang *et al*. used, we predict male adult obesity cases will be averted. Therefore, we took medical costs from a study due to Oster *et al*., which includes obese men and women [49]. Oster *et al*. used NHANES III [50] to estimate the costs associated with hyper-tension, hypercholesterolemia, type 2 diabetes mellitus, cardiovascular disease, and stroke [49]. The age period for averted medical costs was 35 years old until death rather than 40–64 years of age as we would have preferred. If the BMI score is in a category >32.5 *kg*/*m*^2 ^in Oster *et al*., then we considered the person to be obese. Recall that our definition is based on BMI being greater than 30 *kg*/*m*^2 ^. However, this was as close to our definition as possible given the existing literature.

In order to ensure comparability with Wang *et al*., we also considered *NB *and *CER *using parameters for medical costs 40–64 years of age, inclusive. from Gorsky *et al*. (see Table 3) [48]. Because Gorsky *et al*. only estimated medical costs for females, using their estimates necessitated substituting medical costs for females for males [48].

**Table 3:** Net Benefits (NB) and Cost-Effectiveness Ratio (CER) US$ Per *QALY *saved

	Overall		Hispanic Parameters*	
	I	II†	I	II†

Intervention Cost	US$44,039	US$44,039	† US$44,039	US$44,039
Cases Overweight Averted	14.93	14.93	14.93	14.93
*QALY*s Saved	8.55	8.55	8.52	8.52
Medical Costs Averted	US$36,348	US$51,590	US$36,348	US$51,590
Costs of lost labor productivity averted	US$75,816	US$75,816	US$50,929	US$50,929
Cost-effectiveness ratio	US$900	0	US$903	0
Net Benefit	US$68,125	US$83,368	US$43,239	US$58,481

*Uses Hispanic estimates for *QALY*s, labor productivity, and median wages.

† Using female obesity medical costs 40–64 used in Wang *et al*. [35]

### Labor productivity costs (indirect costs)

Equations (5, 6, and 7) in the appendix were used to estimate labor productivity costs. In order to estimate labor productivity costs averted, we estimated the number of sick days missed per year by obese adults in comparison to non-obese adults for persons aged 40–64, inclusive, or from the age of 40 until the person turns 65 years of age. We used median wages to place values on the lost time due to obesity-related illnesses for persons aged 40–64, inclusive. We also estimated the number of lost sick days for the obese and the non-obese using Poisson regression. The model controlled for age, age 40–64, smoking status, Hispanic ethnicity, and gender.

In addition to increased sick days, obese adults also have reduced life expectancy. Therefore, to assume that people aged 40 will live and work until they turn 65 years old would be to over-estimate labor productivity losses averted because more obese 40 year olds will die before 65 than non-obese 40 year olds. Therefore, life expectancy and mortality for obese and non-obese 40-year olds *who die before 65 *were calculated. We also estimated the life expectancy for those alive at 40 who die *before *65 by gender for obese adults and for non-obese adults.

### Data

In order to project lost work days, we used 2002 National Health Interview Survey (NHIS) data. Because of the complex sampling design of the NHIS data, we estimated the model with STATA 7.0©, again using the 'svy' feature. As seen in Table 3, we included overall costs of work-loss estimates and Hispanic costs of work-loss estimates.

Peeters *et al*. created life tables for both men and women by obesity status based on Framingham data [51]. Thus, we were able to project the life expectancy at 40 for an obese person conditional on dying before 65 years of age.

In order to place a value on the sick days averted in our net benefit analysis, we used U.S. Department of Labor, Bureau of Labor Statistics Current Population Survey data [52]. The data are for full-time workers only above 25 years of age for all workers, above 16 years of age for Hispanics. The median wage data is reported by week only. Therefore, in order to estimate the daily wage, the weekly wage was divided by five; in order to calculated the yearly wage, the weekly wage was multiplied by 52.

### Quality-Adjusted Life-Years (*QALY*s)

Equation (4) in the appendix was used to estimate *QALY*s. *QALY*s in our context are the additional quality-adjusted life-years gained through avoiding adult obesity. Activity scales were used in *QALY *to weight, or quality-adjust, years of life that may be added due to the intervention based on questions regarding their activity limitations, if any, and perceived health status [53]. In our study, we estimated scales using the Centers for Disease Control and Prevention's activity scale matrix using 2002 NHIS data. Depending on a person's answer to NHIS survey questions, a health state value is assigned ranging from 0.10 (limited with poor health) up to 1.00 (no limitation with excellent health).

### Data

NHIS survey questions on self-assessed health and activity limitations were used. We again used life tables due to Peeters *et al*. to project the life expectancy at 40 for an obese person [51].

### Sensitivity analysis

In order to determine the extent to which our results are dependent on the parameters we used, sensitivity analysis was conducted for both overall parameters and with parameters for Hispanics. All 48 parameters used in the analysis in Tables 2 and 4 were included in the sensitivity analysis (the Hispanic parameters in the lower part of Table 4 replace the corresponding parameters in the upper part of the table). In order to avoid the problems of the infinite support in the normal distribution, the triangular distribution, which has a finite support, was assumed. The support of the triangular distribution was created from the 95^*th *^percentile confidence intervals of our 48 parameters. We conducted 1,000 independent simulations trials. Each simulation trial draws were made for each of the 48 parameters simultaneously, and *CER *and *NB *calculated (see Table 5). Separate simulations, using the same method as above, were conducted for each of the 48 parameters, holding the other 47 parameters constant.

**Table 4:** Sensitivity Analysis

Variable	Mean	95% Lower Limit	95% Upper Limit
Cases overweight prevented	16.22	15.96	16.48
*QALY*s Saved	7.95	7.82	8.08
Medical Costs Averted	US$39,489	US$38,858	US$40,119
Costs of lost labor productivity averted	US$30,130	US$28,510	US$31,750
Cost-effectiveness ratio	US$1,021	US$900	US$1,143
Net Benefit	US$25,580	US$23,707	US$27,453

Hispanic Parameters			

Cases overweight prevented	16.22	15.96	16.48
*QALY*s Saved	8.00	7.86	8.13
Medical Costs Averted	US$39,489	US$38,858	US$40,119
Costs of lost labor productivity averted	US$21,158	US$20,086	US$22,230
Cost-effectiveness ratio	1,016	895	1,137
Net Benefit	US$16,608	US$15,234	US$17,983

**Table 5:** Parameters Used in the Sensitivity Analysis†

Parameter	Mean	Lower 95^*th *^CL	Upper 95^*th *^CL	Source
*S*_*nm*_	0.872	0.867	0.877	NHIS
*S*_*nf*_	0.859	0.854	0.865	NHIS
*S*_*om*_	0.807	0.800	0.814	NHIS
*S*_*of*_	0.795	0.787	0.802	NHIS
*M*_*nm*_	0.079	0.078	0.081	[51]
*M*_*nf*_	0.066	0.065	0.068	[51]
*M*_*om*_	0.147	0.144	0.149	[51]
*M*_*of*_	0.145	0.142	0.147	[51]
*L*_*nm*_	18.57	15.86	21.30	[51]
*L*_*nf*_	16.94	15.67	18.24	[51]
*L*_*om*_	18.46	13.90	24.36	[51]
*L*_*of*_	16.80	14.15	19.34	[51]
*D*_*nm*_	1.33	1.15	1.50	NHIS
*D*_*nf*_	1.46	1.32	1.61	NHIS
*D*_*om*_	1.88	1.68	2.09	NHIS
*D*_*of*_	2.02	1.83	2.21	NHIS
Wdm* MathType@MTEF@5@5@+=feaafiart1ev1aaatCvAUfKttLearuWrP9MDH5MBPbIqV92AaeXatLxBI9gBaebbnrfifHhDYfgasaacH8akY=wiFfYdH8Gipec8Eeeu0xXdbba9frFj0=OqFfea0dXdd9vqai=hGuQ8kuc9pgc9s8qqaq=dirpe0xb9q8qiLsFr0=vr0=vr0dc8meaabaqaciaacaGaaeqabaqabeGadaaakeaacqWGxbWvdaqhaaWcbaGaemizaqMaemyBa0gabaGaeiOkaOcaaaaa@31A0@	148.8	148.0	149.6	[52]
Wdf∗ MathType@MTEF@5@5@+=feaafiart1ev1aaatCvAUfKttLearuWrP9MDH5MBPbIqV92AaeXatLxBI9gBaebbnrfifHhDYfgasaacH8akY=wiFfYdH8Gipec8Eeeu0xXdbba9frFj0=OqFfea0dXdd9vqai=hGuQ8kuc9pgc9s8qqaq=dirpe0xb9q8qiLsFr0=vr0=vr0dc8meaabaqaciaacaGaaeqabaqabeGadaaakeaacqWGxbWvdaqhaaWcbaGaemizaqMaemOzaygabaGaey4fIOcaaaaa@31A5@	116.8	116.0	117.6	[52]
Wym∗ MathType@MTEF@5@5@+=feaafiart1ev1aaatCvAUfKttLearuWrP9MDH5MBPbIqV92AaeXatLxBI9gBaebbnrfifHhDYfgasaacH8akY=wiFfYdH8Gipec8Eeeu0xXdbba9frFj0=OqFfea0dXdd9vqai=hGuQ8kuc9pgc9s8qqaq=dirpe0xb9q8qiLsFr0=vr0=vr0dc8meaabaqaciaacaGaaeqabaqabeGadaaakeaacqWGxbWvdaqhaaWcbaGaemyEaKNaemyBa0gabaGaey4fIOcaaaaa@31DD@	US$38,688	US$38,484	US$38,892	[52]
Wyf∗ MathType@MTEF@5@5@+=feaafiart1ev1aaatCvAUfKttLearuWrP9MDH5MBPbIqV92AaeXatLxBI9gBaebbnrfifHhDYfgasaacH8akY=wiFfYdH8Gipec8Eeeu0xXdbba9frFj0=OqFfea0dXdd9vqai=hGuQ8kuc9pgc9s8qqaq=dirpe0xb9q8qiLsFr0=vr0=vr0dc8meaabaqaciaacaGaaeqabaqabeGadaaakeaacqWGxbWvdaqhaaWcbaGaemyEaKNaemOzaygabaGaey4fIOcaaaaa@31CF@	US$30,368	US$30,164	US$30,572	[52]

Hispanic Parameters				

*S*_*nm*_	0.855	0.848	0.863	NHIS
*S*_*nf*_	0.842	0.835	0.850	NHIS
*S*_*om*_	0.791	0.782	0.799	NHIS
*S*_*of*_	0.778	0.769	0.787	NHIS
*D*_*nm*_	1.26	1.04	1.48	NHIS
*D*_*nf*_	1.40	1.18	1.62	NHIS
*D*_*om*_	1.82	1.58	2.06	NHIS
*D*_*of*_	1.96	1.72	2.20	NHIS
Wdm* MathType@MTEF@5@5@+=feaafiart1ev1aaatCvAUfKttLearuWrP9MDH5MBPbIqV92AaeXatLxBI9gBaebbnrfifHhDYfgasaacH8akY=wiFfYdH8Gipec8Eeeu0xXdbba9frFj0=OqFfea0dXdd9vqai=hGuQ8kuc9pgc9s8qqaq=dirpe0xb9q8qiLsFr0=vr0=vr0dc8meaabaqaciaacaGaaeqabaqabeGadaaakeaacqWGxbWvdaqhaaWcbaGaemizaqMaemyBa0gabaGaeiOkaOcaaaaa@31A0@	100.2	98.1	102.3	[52]
Wdf∗ MathType@MTEF@5@5@+=feaafiart1ev1aaatCvAUfKttLearuWrP9MDH5MBPbIqV92AaeXatLxBI9gBaebbnrfifHhDYfgasaacH8akY=wiFfYdH8Gipec8Eeeu0xXdbba9frFj0=OqFfea0dXdd9vqai=hGuQ8kuc9pgc9s8qqaq=dirpe0xb9q8qiLsFr0=vr0=vr0dc8meaabaqaciaacaGaaeqabaqabeGadaaakeaacqWGxbWvdaqhaaWcbaGaemizaqMaemOzaygabaGaey4fIOcaaaaa@31A5@	88.6	86.9	90.3	[52]
Wvm∗ MathType@MTEF@5@5@+=feaafiart1ev1aaatCvAUfKttLearuWrP9MDH5MBPbIqV92AaeXatLxBI9gBaebbnrfifHhDYfgasaacH8akY=wiFfYdH8Gipec8Eeeu0xXdbba9frFj0=OqFfea0dXdd9vqai=hGuQ8kuc9pgc9s8qqaq=dirpe0xb9q8qiLsFr0=vr0=vr0dc8meaabaqaciaacaGaaeqabaqabeGadaaakeaacqWGxbWvdaqhaaWcbaGaemODayNaemyBa0gabaGaey4fIOcaaaaa@31D7@	US$26,058	US$25,508	US$26,609	[52]
Wyf∗ MathType@MTEF@5@5@+=feaafiart1ev1aaatCvAUfKttLearuWrP9MDH5MBPbIqV92AaeXatLxBI9gBaebbnrfifHhDYfgasaacH8akY=wiFfYdH8Gipec8Eeeu0xXdbba9frFj0=OqFfea0dXdd9vqai=hGuQ8kuc9pgc9s8qqaq=dirpe0xb9q8qiLsFr0=vr0=vr0dc8meaabaqaciaacaGaaeqabaqabeGadaaakeaacqWGxbWvdaqhaaWcbaGaemyEaKNaemOzaygabaGaey4fIOcaaaaa@31CF@	US$23,026	US$22,585	US$23,466	[52]

Note that the probabilities in Table 2 are also included in the sensitivity analysis.

*Calculated from weekly salaries.

[51]. Peeters A, Barendregt JJ, Willekens F, Mackenbach JP, Mamun AA, Bonneux L, NEDCOM tNE, of Morbidity Research Group DC: Obesity in adulthood and its consequences for life expectancy: a life-table analysis. Annals of Internal Medicine 2003, 138:24–31.

[52] US Department of Labor: Highlights of Women's Earnings in 2003. Tech. rep., Bureau of Labor Statistics 2004.

## Results

The results are shown below in Table 3. As noted earlier, the generally accepted conservative threshold is US$30,000 per *QALY *gained [43-45]. Notice that when overall parameters are used and lifetime medical costs are used, the *CER *was US$900 in 2004 dollars. This indicates that the intervention is cost-effective. When Hispanic parameters are used, the *CER *remains very low at US$903.

*NB *was also quite high, meaning that CATCH is a good investment of public resources. In this case, using Hispanic parameters for *QALY*s, labor productivity, and median wages reduced the *NB *by approximately one-third. This is mainly due to the lower wages that Hispanics earn. When the higher medical costs used in Wang *et al*. [35] are used, the *NB *rose to US$83,368.

From our calculations based on Oster *et al*. [49], the *lifetime *medical cost differential for obese males 35–64 years old and non-obese males was US$9,716 while the difference for an obese woman 35–64 years old and a non-obese woman was US$11,086 [49]. In present value terms, using a 3% interest rate, the difference in lifetime medical costs for obese men versus non-obese men was US$4,123 and for women the difference was US$4,704, as seen in Table 3.

The sensitivity analysis revealed that in all cases, the intervention remained cost-effective and net beneficial. To ensure the robustness of our results, we also varied the rate of discount. Not surprisingly, the greater the future was discounted, the lower the *NB *and *CER*. Still, even when the rate of discount was five percent, CATCH remained cost-effective and net beneficial.

## Discussion

There is a dearth of economic research on the value of school-based health promotions for the Hispanic population. The results here are the first to indicate that these programs are net beneficial and cost-effective. This is despite the lower wages earned by Hispanics, which means that the value of averted labor costs is lower.

CATCH compares favorably to alternative school-based health promotions. Wang *et al*. [35] estimated Planet Health's cost-effectiveness ratio to be US$5,166 per *QALY *(2004 dollars). When the medical costs used by Wang *et al*. [35] to evaluate Planet Health are used to evaluate CATCH (recall that this necessitated substituting female medical costs for males), the *CER *of CATCH decreased to US$0 for both the overall estimate and estimate based on Hispanic parameters (This is referred to as a cost saving result). However, note that Planet Health is cost-effective.

Wang *et al*. [35] estimated Planet Health's cost-effectiveness ratio to be US$8,776 (2004 dollars). Although Planet health is clearly net beneficial, it is less so than CATCH. This is mainly due to the fact that in the CATCH trial, there were averted overweight and at-risk boys which lead to averted obese males. Therefore, because males earn higher wages than females, the *NB*s were higher for males

## Conclusion

This is the second study of the cost-effectiveness of a school-based intervention for programs targeting childhood obesity. The *CER *for CATCH was US$900. Further, when we used the medical costs used in Wang et al. (see II. Cost-effectiveness ratio in Table 3) [35], the *CER *decreased to US$0. Both estimates are well underneath the US$30,000 threshold value [43-45] of a human life-year. Our sensitivity analysis reveals that the results are robust.

With the growth of the Hispanic population in the United States, school-based overweight programs that are cost-effective for this population will be increasingly important. *CER *was US$903 when Hispanic parameters were used. The *N B *was US$69,764. Therefore, this study confirms that school-based overweight programs such as CATCH are both cost-effective and net beneficial in Hispanic populations.

Wang et al. estimated Planet Health's cost-effectiveness ratio to be US$4,305 per *QALY *(US$5,166 in 2004 dollars) However, note that there were many different parameters used in our study, necessitated by the fact that the CATCH trial was successful in curbing the prevalence of both boys and girls at-risk for overweight and overweight, whereas Planet Health only curbed girl overweight prevalence. Both programs are easily under any *CER *threshold.

There are limitations of this study. First, we are forced to project of adult obesity cases averted. Future medical technology or other changes mean that obesity rates may decline in the future, our sensitivity analysis allows to vary. One of the strengths of our approach is that our results are robust to changes in our estimates.

A second limitation is the lack of availability of medical cost estimates for obese males 40–64.

Despite the limitations of the study, the results show that an expansion of CATCH and/or similar school-based health promotion interventions would aid in limiting overweight prevalence in a cost-effective and net beneficial manner. Thus, public health efforts should focus on the implementation of school-based programs as an effective means of prevention of overweight, by advocating policy efforts such as mandates for health promotion in Texas, as well as convincing educators and administrators that their school-based obesity prevention programs are as essential to society as their academic programs.

## Appendix

### Additional Formulae

#### Quality Adjusted Life-Years

Q={∑iMniSni[1r−1r(1+r)Lni]−∑iMoiSoi[1r−1r(1+r)Loi]+[∑i(1−Mni)Sni−(1−Moi)Soi][1r−1r(1+r)25]}(1+r)29     (4)
 MathType@MTEF@5@5@+=feaafiart1ev1aaatCvAUfKttLearuWrP9MDH5MBPbIqV92AaeXatLxBI9gBaebbnrfifHhDYfgasaacH8akY=wiFfYdH8Gipec8Eeeu0xXdbba9frFj0=OqFfea0dXdd9vqai=hGuQ8kuc9pgc9s8qqaq=dirpe0xb9q8qiLsFr0=vr0=vr0dc8meaabaqaciaacaGaaeqabaqabeGadaaakeaacqWGrbqucqGH9aqpdaWcaaqaaiabcUha7naalaaabaWaaabeaeaacqWGnbqtdaWgaaWcbaGaemOBa4MaemyAaKgabeaakiabdofatnaaBaaaleaacqWGUbGBcqWGPbqAaeqaaOGaei4waS1aaSaaaeaacqaIXaqmaeaacqWGYbGCaaGaeyOeI0YaaSaaaeaacqaIXaqmaeaacqWGYbGCcqGGOaakcqaIXaqmcqGHRaWkcqWGYbGCcqGGPaqkdaahaaWcbeqaaiabdYeamnaaBaaameaacqWGUbGBcqWGPbqAaeqaaaaaaaGccqGGDbqxaSqaaiabdMgaPbqab0GaeyyeIuoakiabgkHiTmaaqababaGaemyta00aaSbaaSqaaiabd+gaVjabdMgaPbqabaGccqWGtbWudaWgaaWcbaGaem4Ba8MaemyAaKgabeaakiabcUfaBnaalaaabaGaeGymaedabaGaemOCaihaaiabgkHiTmaalaaabaGaeGymaedabaGaemOCaiNaeiikaGIaeGymaeJaey4kaSIaemOCaiNaeiykaKYaaWbaaSqabeaacqWGmbatdaWgaaadbaGaem4Ba8MaemyAaKgabeaaaaaaaOGaeiyxa0faleaacqWGPbqAaeqaniabggHiLdaakeaacqGHRaWkcqGGBbWwdaaeqaqaaiabcIcaOiabigdaXiabgkHiTiabd2eannaaBaaaleaacqWGUbGBcqWGPbqAaeqaaOGaeiykaKIaem4uam1aaSbaaSqaaiabd6gaUjabdMgaPbqabaGccqGHsislcqGGOaakcqaIXaqmcqGHsislcqWGnbqtdaWgaaWcbaGaem4Ba8MaemyAaKgabeaakiabcMcaPiabdofatnaaBaaaleaacqWGVbWBcqWGPbqAaeqaaOGaeiyxa0Laei4waS1aaSaaaeaacqaIXaqmaeaacqWGYbGCaaGaeyOeI0YaaSaaaeaacqaIXaqmaeaacqWGYbGCcqGGOaakcqaIXaqmcqGHRaWkcqWGYbGCcqGGPaqkdaahaaWcbeqaaiabikdaYiabiwda1aaaaaGccqGGDbqxaSqaaiabdMgaPbqab0GaeyyeIuoaaaGccqGG9bqFaeaacqGGOaakcqaIXaqmcqGHRaWkcqWGYbGCcqGGPaqkdaahaaWcbeqaaiabikdaYiabiMda5aaaaaaaaa@A294@

where

*S*_*ni *_= Activity scale for non-obese by gender

*S*_*oi *_= Activity scale for obese by gender

*M*_*ni *_= Death probability 40–64 for non-obese by gender

*M*_*oi *_= Death probability 40–64 for obese by gender

*L*_*ni *_= Life expectancy for non-obese 40 who die by 65 by gender

*L*_*oi *_= Life expectancy for obese 40 who die by 65 by gender

*r *= the rate of discount

#### Productivity

*B*1 + *B*2 = *B*     (5)

B1=Wdi{MoiDoi[1r−1r(1+r)Loi]−MniDni[1r−1r(1+r)Lni]+[(1−Moi)Doi−(1−Mni)Dni][1r−1r(1+r)25]}(1+r)29     (6)
 MathType@MTEF@5@5@+=feaafiart1ev1aaatCvAUfKttLearuWrP9MDH5MBPbIqV92AaeXatLxBI9gBaebbnrfifHhDYfgasaacH8akY=wiFfYdH8Gipec8Eeeu0xXdbba9frFj0=OqFfea0dXdd9vqai=hGuQ8kuc9pgc9s8qqaq=dirpe0xb9q8qiLsFr0=vr0=vr0dc8meaabaqaciaacaGaaeqabaqabeGadaaakeaacqWGcbGqcqaIXaqmcqGH9aqpdaWcaaqaaiabdEfaxnaaBaaaleaacqWGKbazcqWGPbqAaeqaaOGaei4EaS3aaSaaaeaacqWGnbqtdaWgaaWcbaGaem4Ba8MaemyAaKgabeaakiabdseaenaaBaaaleaacqWGVbWBcqWGPbqAaeqaaOGaei4waS1aaSaaaeaacqaIXaqmaeaacqWGYbGCaaGaeyOeI0YaaSaaaeaacqaIXaqmaeaacqWGYbGCcqGGOaakcqaIXaqmcqGHRaWkcqWGYbGCcqGGPaqkdaahaaWcbeqaaiabdYeamnaaBaaameaacqWGVbWBcqWGPbqAaeqaaaaaaaGccqGGDbqxcqGHsislcqWGnbqtdaWgaaWcbaGaemOBa4MaemyAaKgabeaakiabdseaenaaBaaaleaacqWGUbGBcqWGPbqAaeqaaOGaei4waS1aaSaaaeaacqaIXaqmaeaacqWGYbGCaaGaeyOeI0YaaSaaaeaacqaIXaqmaeaacqWGYbGCcqGGOaakcqaIXaqmcqGHRaWkcqWGYbGCcqGGPaqkdaahaaWcbeqaaiabdYeamnaaBaaameaacqWGUbGBcqWGPbqAaeqaaaaaaaGccqGGDbqxcqGHRaWkaeaacqGGBbWwcqGGOaakcqaIXaqmcqGHsislcqWGnbqtdaWgaaWcbaGaem4Ba8MaemyAaKgabeaakiabcMcaPiabdseaenaaBaaaleaacqWGVbWBcqWGPbqAaeqaaOGaeyOeI0IaeiikaGIaeGymaeJaeyOeI0Iaemyta00aaSbaaSqaaiabd6gaUjabdMgaPbqabaGccqGGPaqkcqWGebardaWgaaWcbaGaemOBa4MaemyAaKgabeaakiabc2faDjabcUfaBnaalaaabaGaeGymaedabaGaemOCaihaaiabgkHiTmaalaaabaGaeGymaedabaGaemOCaiNaeiikaGIaeGymaeJaey4kaSIaemOCaiNaeiykaKYaaWbaaSqabeaacqaIYaGmcqaI1aqnaaaaaOGaeiyxa0faaiabc2ha9bqaaiabcIcaOiabigdaXiabgUcaRiabdkhaYjabcMcaPmaaCaaaleqabaGaeGOmaiJaeGyoaKdaaaaaaaa@9D2F@

B2=Wyi{Mni[1r−1r(1+r)Lni]−Moi[1r−1r(1+r)Loi]+(Moi−Mni)[1r−1r(1+r)25]}(1+r)29     (7)
 MathType@MTEF@5@5@+=feaafiart1ev1aaatCvAUfKttLearuWrP9MDH5MBPbIqV92AaeXatLxBI9gBaebbnrfifHhDYfgasaacH8akY=wiFfYdH8Gipec8Eeeu0xXdbba9frFj0=OqFfea0dXdd9vqai=hGuQ8kuc9pgc9s8qqaq=dirpe0xb9q8qiLsFr0=vr0=vr0dc8meaabaqaciaacaGaaeqabaqabeGadaaakeaacqWGcbGqcqaIYaGmcqGH9aqpdaWcaaqaaiabdEfaxnaaBaaaleaacqWG5bqEcqWGPbqAaeqaaOGaei4EaSNaemyta00aaSbaaSqaaiabd6gaUjabdMgaPbqabaGccqGGBbWwdaWcaaqaaiabigdaXaqaaiabdkhaYbaacqGHsisldaWcaaqaaiabigdaXaqaaiabdkhaYjabcIcaOiabigdaXiabgUcaRiabdkhaYjabcMcaPmaaCaaaleqabaGaemitaW0aaSbaaWqaaiabd6gaUjabdMgaPbqabaaaaaaakiabc2faDjabgkHiTiabd2eannaaBaaaleaacqWGVbWBcqWGPbqAaeqaaOGaei4waS1aaSaaaeaacqaIXaqmaeaacqWGYbGCaaGaeyOeI0YaaSaaaeaacqaIXaqmaeaacqWGYbGCcqGGOaakcqaIXaqmcqGHRaWkcqWGYbGCcqGGPaqkdaahaaWcbeqaaiabdYeamnaaBaaameaacqWGVbWBcqWGPbqAaeqaaaaaaaGccqGGDbqxcqGHRaWkcqGGOaakcqWGnbqtdaWgaaWcbaGaem4Ba8MaemyAaKgabeaakiabgkHiTiabd2eannaaBaaaleaacqWGUbGBcqWGPbqAaeqaaOGaeiykaKIaei4waS1aaSaaaeaacqaIXaqmaeaacqWGYbGCaaGaeyOeI0YaaSaaaeaacqaIXaqmaeaacqWGYbGCcqGGOaakcqaIXaqmcqGHRaWkcqWGYbGCcqGGPaqkdaahaaWcbeqaaiabikdaYiabiwda1aaaaaGccqGGDbqxcqGG9bqFaeaacqGGOaakcqaIXaqmcqGHRaWkcqWGYbGCcqGGPaqkdaahaaWcbeqaaiabikdaYiabiMda5aaaaaaaaa@853F@

where

*D*_*ni *_= Missed days for the non-obese by gender

*D*_*oi *_= Missed days for the obese by gender

*W*_*di *_= Daily wage by gender

*W*_*yi *_= Yearly wage by gender.
